# Association of Air Pollution and Mortality of Acute Lower Respiratory Tract Infections in Shenyang, China: A Time Series Analysis Study

**Published:** 2018-09

**Authors:** Jie GUO, Mingyue MA, Chunling XIAO, Chunqing ZHANG, Jianping CHEN, Hong LIN, Yiming DU, Min LIU

**Affiliations:** 1. Dept. of Pathogenic Biology, Shenyang Medical College, Shenyang, China; 2. Key Laboratory of Environmental Pollution and Microecology of Liaoning Province, Shenyang, China; 3. Shenyang Center for Disease Control and Prevention, Shenyang, China; 4. Shenyang Environmental Monitoring Center Station, Shenyang, China

**Keywords:** Air pollution, Acute lower respiratory tract infection, Time series study

## Abstract

**Background:**

We aimed to evaluate the risk factors of the daily mortality associated with air pollution causing acute lower respiratory tract infections.

**Methods:**

We applied a short time series analysis to the air pollution record, meteorological data and 133 non-accidental death data in Shengyang, China, in 2013–2015. After controlling the seasonality, day of week and weather conditions, the group employed an over-dispersed Possion generalized addictive model to discuss the associations among different variables, then performed the stratified analysis according to age, gender, and season.

**Results:**

Mean concentrations of particulate matter with aerodynamic diameters of < 10 μm (PM_10_) and < 2.5 μm (PM_2.5_), sulfur dioxide (SO_2_), nitrogen dioxide (NO_2_), and ozone (O_3_) were 122.4, 74.8, 79.4, 47.7, and 86.2 μg/m^3^, respectively. An increase of 10 μg/m^3^ in the 8-day moving average concentrations of PM_10_, PM_2.5_, SO_2_, NO_2_, and O_3_ corresponded to 0.18% (95% confidence interval [CI]: 0.10%, 0.26%), 0.21% (95% CI: 0.11%, 0.31%), 0.16% (95% CI: 0.04%, 0.30%), 0.43% (95% CI: 0.07%, 0.90%), and 0.10% (95% CI: −0.08%, 0.31%) increase in the daily mortality. The effects of air pollution lasted 9 days (lag 0–8), and they were more statistically significant in the elderly than in other age groups.

**Conclusion:**

These findings clarified the burden of air pollution on the morbidity of acute lower respiratory tract infections and emphasized the urgency of the control and prevention of air pollution and respiratory diseases in China.

## Introduction

With rapid economic growth and urbanization, industrialization and air pollution have posed threats to human health and have raised substantial attention worldwide. The adverse effects of air pollution on health have also emerged as a remarkable public concern (1, 2). Long- and short-term exposure to air pollution are associated with acute and chronic death risks. Air pollution is a complex phenomenon that involves gaseous compounds, including sulfur dioxide (SO_2_), nitrogen oxides, ozone (O_3_), carbon monoxide, and a heterogeneous mixture of gaseous pollutants and particulate matter (PM) that may vary in composition depending on geographical areas and meteorological conditions (3, 4). Mortality is one of the effects of exposure to air pollutants (5). “The risk of death associated with respiratory problems caused by exposure to air pollutants is significantly higher than that of cardiovascular diseases in 61% of cases, with an average relative risk of 6%–10%” (6, 7).

Approximately 120–156 million cases of acute lower respiratory tract infections (ALRIs) are reported annually worldwide, and 1.4 million of these cases result in death. ALRIs are caused by various infective agents, such as *Streptococcus pneumoniae* and respiratory syncytial virus (8). “Many factors determine whether a contact with an etiologic agent will trigger a severe episode of ALRI, and whether such episode will cause death” (9). These factors may be related to patients (e.g., age, sex, underlying diseases), diseases (e.g., type of infection), environment, patient’s family and their socio-economic status, health system, and type of care (9, 10). “More than 95% of the reported deaths occur in low- and middle-income countries” (11–13).

Studies on the impacts of air pollution on death have been mostly performed in Beijing, Shanghai, and Guangzhou, but few studies have used data from northeast Shenyang to assess the effects of air pollution on human health.

Therefore, this study further evaluated the impact of air pollution on daily mortality by conducting a time series analysis based on a large individual dataset of Shenyang, China, to facilitate an evidence-based policy-making and resource allocation.

## Methods

### Data collection

Shenyang is located in the northeastern plains of China. It is one of the political, economic, cultural, transportation, finance, information, tourism, and industrial centers and one of the historical and cultural cities in northeast China. In 2015, the city had a permanent population of 12.78 million. Shenyang has a temperate semi-humid continental climate affected by monsoon and four seasons. In this area, winter is cold and dry, whereas summer is moist and rainy. Temperature changes in various seasons. However, economic and industrial development in Shenyang have led to serious air pollution problems that might have affected human health.

### Mortality Data

Three diseases that are sensitive to the acute onset of death events were selected in this study. Anonymous data on cases of non-accidental deaths between January 1, 2013 and December 31, 2015 were obtained from the Shenyang Municipal Center for Disease Control and Prevention. The data were restricted to registered residents and composed of 133 non-accidental deaths (66 males, 67 females) in the three study years. The mortality data were encoded according to the 10th revision of the International Statistical Classification of Diseases and Related Health Problems, and 133 of deaths were from respiratory diseases (ICD 10^th^ revision: J20 to J22). Gender and age at death, among other variables, were also documented.

### Exposure Data

**Fig. 1:**
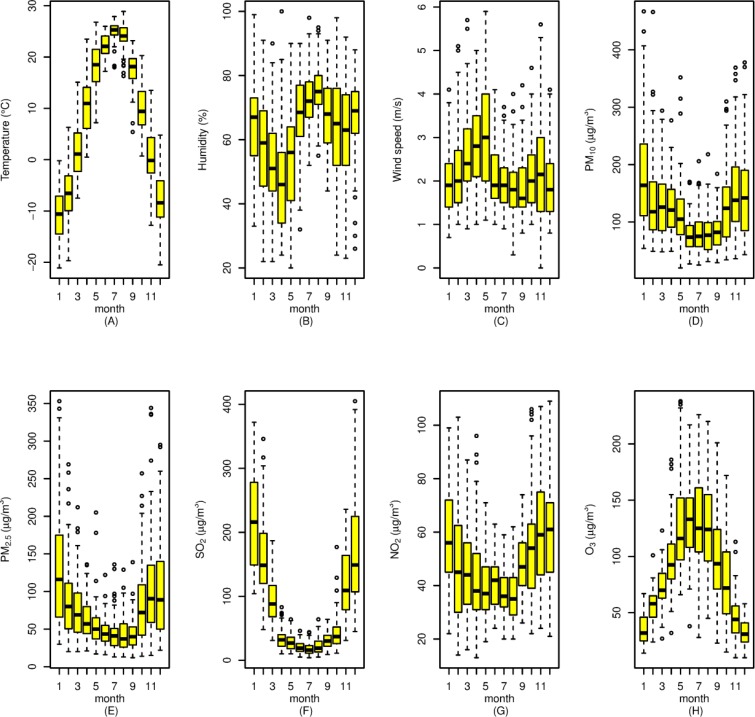
Boxplots of monthly temperature, relative humidity, wind speed, PM_10_, PM_2.5_, SO_2_, NO_2_, and O_3_ in Shenyang, China, in 2013–2015

We collected daily data on air pollutants from Shenyang Environmental Monitoring Center during the same study period, the concentrations of PM_10_, PM_2.5_, NO_2_ and SO_2_ were monitored consecutively at 11 monitoring sites that cover all districts and counties of Shenyang, including both urban and suburban areas. The pollutants concentrations were measured according to the Chinese National Ambient Air Quality Standard (14). The pollutant concentrations were recorded on an hourly basis from which the daily average concentrations of pollutants for individual monitoring sites and the average levels for the entire Shenyang City were derived. Meteorological data, including daily mean temperature, relative humidity, air pressure, and wind speed, were obtained from the data provided by the Shenyang Meteorological Bureau (Fig. 1).

### Statistical analyses

Time series regression model analysis was used to investigate the short-term associations between air pollution and death caused by ALRI. Time series (dynamic series) are routinely analyzed in statistical methods. Thus, time series (dynamic sequence) analysis a routine method used to investigate the acute health effects of air pollution in terms of mortality data because it can be well controlled by time variables, such as seasonal variables, and other confounding factors. Given that the daily mortality data of the ALRIs were approximately Poisson distributed, the Poisson generalized additive model was used for data analysis. The daily mortality was considered the dependent variable in the following model formula:

$$\mathrm{Log}\left[E\left(Y_{t}\right)\right]=\mathrm{Intercept}+\alpha T_{t,l}\left(Z\right)+\mathrm{ns}\left(\mathrm{TM},3\right)+\mathrm{ns}\left(\mathrm{RH},3\right)+\mathrm{ns}\left(\mathrm{WS},3\right)+\mathrm{ns}\left(\mathrm{AP},3\right)+\mathrm{ns}\left(\mathrm{time},\mathrm{df}\right)+\beta \mathrm{DOW}$$

where *E* (*Yt*) is the expected mortality of ALRIs on day *t*, ns is a natural cubic smooth function, *t* is the day of observation, *l* is the number of lag days, *Z* is the individual air pollutants, *T_t,l_* is a function of MA(Moving Average) of air pollutants, *α* is the coefficients for *T_t,l_*. Degrees of freedom for the Model were chosen based on Akaike Information Criterion (1). *TM* is the temperature, *RH* is the relative humidity, *WS* is the wind speed, and *AP* is the air pressure. *Dow* is the day of week, which was used as a dummy variable, and *β* is the coefficients for *Dow*.

To investigate the lag pattern of air pollution, potentially delayed and cumulative associations were estimated. We first examined the delayed associations using a single day lag (from lag0 to lag7), as previous studies showed the lag effects of particulate matter were strongest within 7 days (16, 17). Then the cumulative associations were estimated using the moving average over the lag periods from lag01 (moving average concentrations of day0 and day1) to lag07 (moving average concentrations of day0 to day7). Considering the air pollution effect of 8-day moving average concentrations from day 0 to day 7 (lag07) is strongest, so we use lag07 in our main analysis. Stratified analyses were conducted according to sex, age, and seasons to identify the populations that were potentially sensitive to air pollution. Data was stratified by season (defined as warm season, May–October; and cold season, November–April). We controlled for the long-term time trend with a natural cubic regression spline with eight degrees of freedom (df) per year (15), and 4 df per season per year for season-stratified analysis. We also controlled for day of the week with indicator variables, and for daily temperature, relative humidity, air pressure, and wind speed with a natural cubic spline with three df for each. We checked the autocorrelations of residuals and seasons by using partial autocorrelation functions (PACF) for the aforementioned model. The PACF plot revealed no significant autocorrelation of the model residuals, indicating that the underlying model was appropriate. All of the models were established in *R* software version 3.1.3 with the “mgcv” package.

## Results

The mortality of the ALRIs was 133 deaths. Of these cases, 90% occurred among those aged above 65 years in the study period (1905 days). Table 1 shows the daily air pollutants, the meteorological conditions, and the number of deaths of the ALRIs. The number of deaths and the air pollutant level were higher in cool seasons than in warm seasons, and both outcomes displayed a seasonal trend (Table 1). The average concentrations of PM_10_, PM_2.5_, SO_2_, NO_2_, and O_3_ were 122.4, 74.8, 79.4, 47.7, and 86.2 μg/m^3^, respectively. Their respective air quality indexes of the World Health Organization for each were 20, 10, 20, 40, and 100 μg/m^3^ (4). In general, the five airborne Spearman coefficients ranging from 0.55 to 0.91 were significantly correlated. The five air pollutants and meteorological parameters were related to one another (Table 2).

**Table 1:** Statistical observations of daily air pollutants, meteorological conditions, and daily mortality in Shenyang, China, in 2013–2015

***Variables***	***All Seasons***		***Cold Season***		***Warm Season***	
	***Mean***	***Standard deviation***	***Mean***	***Standard deviation***	***Mean***	***Standard deviation***
Air pollutants concentrations (24-h average, μg/m^3^)						
PM_10_ (μg/m^3^)	122.4	81.3	148.9	90.5	95.3	55.6
PM_2.5_ (μg/m^3^)	74.8	61.8	92.9	61.6	53.3	41.1
SO_2_(μg/m^3^)	79.4	81.1	135.6	88.0	26.8	17.3
NO_2_ (μg/m^3^)	47.7	18.2	51.9	20.8	42.9	14.0
O_3_(μg/m^3^)	86.2	47.7	58.1	30.2	115.5	43.9
Weather conditions (24-h average)						
Temperature (°C)	8.8	13.2	−1.9	9.2	19.5	6.1
Relative humidity (%)	62.8	15.6	57.5	15.7	66.9	14.3
Air pressure (Pa)	1010.1	9.6	1015.8	7.2	1004.0	7.3
Wind speed (m/s)	2.2	0.9	2.3	1.0	2.2	0.9
Daily death counts (No. of deaths)						
Male	0.06	0.26	0.07	0.28	0.05	0.23
Female	0.06	0.25	0.07	0.26	0.05	0.24
0–65	0.02	0.12	0.02	0.12	0.02	0.13
65<	0.21	0.76	0.24	0.85	0.19	0.66
Total	0.12	0.37	0.14	0.40	0.11	0.33

**Table 2:** Spearman correlation between air pollutants and meteorological conditions in Shenyang, China, in 2013–2015

***Index***	***SO_2_***	***NO_2_***	***PM_10_***	***PM_2.5_***	***O_3_***	***Wind speed***	***Air pressure***	***Mean temperature***	***Relative humidity***
SO_2_	1	0.554^**^	0.647^**^	0.673^**^	−0.644^**^	−0.092^**^	0.738^**^	−0.800^**^	−0.116^**^
NO_2_	-	1	0.612^**^	0.664^**^	−0.356^**^	−0.432^**^	0.407^**^	−0.327^**^	0.068^*^
PM_10_	-	-	1	0.908^**^	−0.204^**^	0.075^*^	0.384^**^	−0.358^**^	−0.039
PM_2.5_	-	-	-	1	−0.305^**^	−0.174^**^	0.442^**^	−0.404^**^	0.127^**^
O_3_	-	-	-	-	1	0.163**	−0.669	0.818**	−0.093^**^
Wind speed	-	-	-	-	-	1	−0.122^**^	0.034	−0.352^**^
Air pressure	-	-	-	-	-	-	1	−0.838^**^	−0.212^**^
Mean temperature	-	-	-	-	-	-	-	1	0.184^**^
Relative humidity	-	-	-	-	-	-	-	-	1

All correlation coefficients not equal to 1 were statistically significant at *P*<0.01

**Fig. 2:**
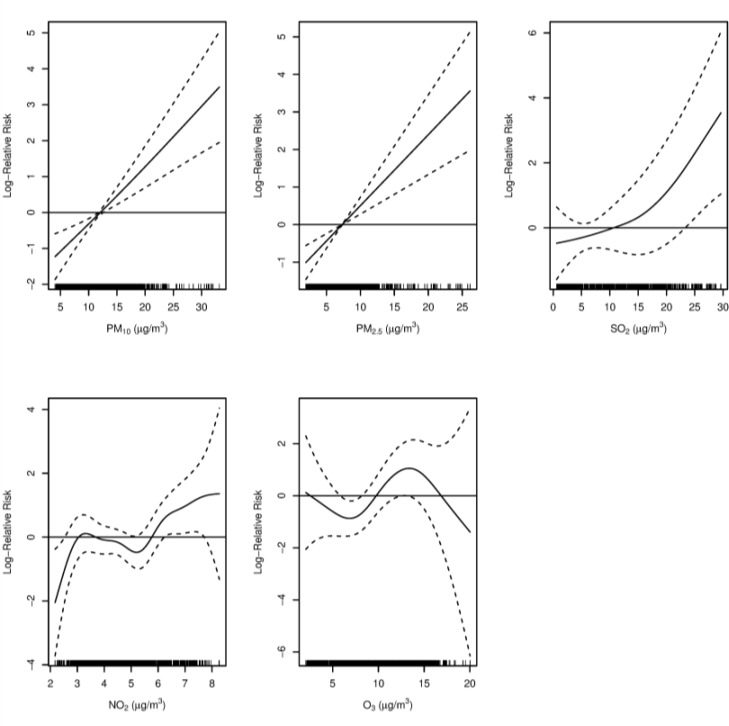
Association of air pollutants (lag 07) with an increase in the relative risk of mortality in Shenyang, China, in 2013–2015. X-axis is the air pollutants concentrations at lag 07. The solid lines indicate the estimated mean percentage of change in daily death ALRIs, and the dotted lines represent twice the standard error

**Fig. 3:**
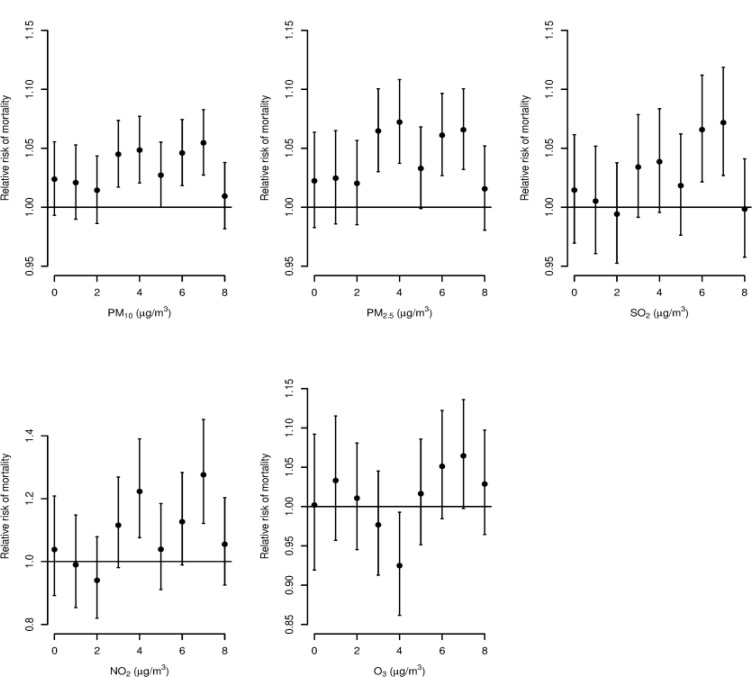
Association of a 10μg/m^3^ increase in PM_10_, PM_2.5_, SO_2_, NO_2_ and O_3_ with an increase in the relative risk of mortality according to single-pollutant models at different lag days in Shenyang, China, in 2013–2015, with adjustments for seasonality, day of the week, temperature, relative humidity, air pressure, and wind speed

Fig. 2 shows the association of death caused by ALRIs with air pollution that lasted 9 days (lag 0–8). Table 3 and Fig. 3 summarize the moving 8-day averaged concentrations of PM_10_, PM_2.5_, SO_2_, NO_2_ and O_3_ cumulative association with an increase of 10 μg/m^3^ pollutants on daily mortality of ALRIs.

**Table 3:** Association of a 10μg/m^3^ increase with the increase in non-accidental deaths due to air pollutants (lag 07) in Shenyang, China, in 2013–2015 according to single-, two-pollutant models

***Model***	***Pollutant***	***Percentage increase in death (95% CI)***
Single pollutant model	PM_10_	0.18(0.10, 0.26)
	SO_2_+PM_10_	0.18(0.08, 0.28)
Multi - pollutant model	NO_2_+PM_10_	0.22(0.11, 0.34)
	O_3_+PM_10_	0.17(0.09, 0.26)
Single pollutant model	PM_2.5_	0.21(0.11, 0.31)
	SO_2_+PM_2.5_	0.21(0.10, 0.34)
Multi - pollutant model	NO_2_+PM_2.5_	0.28(0.14, 0.44)
	O_3_+PM_2.5_	0.20(0.11, 0.31)
Single pollutant model	SO_2_	0.16(0.04, 0.30)
	PM_10_+SO_2_	−0.01(−0.12, 0.14)
	NO_2_+SO_2_	0.12(−0.04, 0.30)
Multi - pollutant model	PM_2.5_+SO_2_	−0.01(−0.13, 0.14)
	O_3_+SO_2_	0.16(0.04, 0.30)
Single pollutant model	NO_2_	0.43(0.07, 0.90)
	SO_2_+NO_2_	0.16(−0.22, 0.73)
	PM_10_+NO_2_	−0.20(−0.45, 0.17)
Multi - pollutant model	PM_2.5_+NO_2_	−0.26(−0.51, 0.11)
	O_3_+NO_2_	0.46(0.10, 0.96)
Single pollutant model	O_3_	0.10(−0.08, 0.31)
	PM_10_+O_3_	0.02(−0.14, 0.22)
	PM_2.5_+O_3_	0.05(−0.12, 0.25)
Multi - pollutant model	SO_2_+O_3_	0.10(−0.08, 0.31)
	NO_2_+O_3_	0.13(−0.05, 0.35)

The analyses were adjusted for seasonality, day of the week, temperature, relative humidity, air pressure, and wind speed

In brief, in the single-pollutant model, an increase of 10 μg/m^3^ in the moving 8-day averaged concentrations of PM_10_, PM_2.5_, SO_2_, NO_2_, and O_3_ was associated with a 0.18% (95% confidence interval [CI]: 0.10%, 0.26%), 0.21% (95% CI: 0.11%, 0.31%), 0.16% (95% CI: 0.04%, 0.30%), 0.43% (95% CI: 0.07%, 0.90%), and 0.10% (95% CI: −0.08%, 0.31%) increase in daily mortality. In the two-pollutant models, the estimates for associations with PM (PM_10_ and PM_2.5_) markedly increased when gaseous pollutants (SO_2_ and NO_2_), particularly NO_2_, were introduced to the model. The estimates for the associations with NO_2_ were increased and PM_10_ and PM_2.5_ decrease when O_3_ were added. The inclusion of PM_10_, PM_2.5_, or NO_2_ in the model considerably correlated the estimated association with O_3_.

Table 4 shows the results of the stratified analysis by gender, age, and season. Seasonal variations were observed in the effects of air pollutants on the total mortality of the acute lower respiratory tract infections. The effects of PM were statistically significant in the cold season but not in the warm season. The estimated effects of PM_10_ and PM_2.5_ on the mortality of the ALRIs were the highest in the cold season, and such effects corresponded to 0.15% (95% CI: 0.02%, 0.29%), and 0.17% (95% CI: 0.03%, 0.33%) increase in the mortality risk of the ALRIs for every 10 μg/m^3^ increase in PM_10_ and PM_2.5_ at moving 8-day averaged in the cold season. By contrast, these effects were not significant in the warm season. Gender and age could modify the association between the exposure to PM_10_, PM_2.5_, SO_2_, NO_2_, and O_3_ and the mortality of the ALRIs. Significantly strong associations were observed between air pollutants and mortality among people aged 65 years or above and female. The effect of NO_2_ on females was significantly stronger than that on males.

**Table 4:** Association of a 10μg/m^3^ increase in air pollutants (lag 07) with the increase in the non-accidental deaths in Shenyang, China, in 2013–2015 according to pollutant models stratified by sex, age, and season

***Group***		***All***	***Sex***		***Age***	
			***Male***	***Female***	***≤65years***	***>65years***
All seasons	PM_10_	0.18(0.10, 0.26)	0.10(0.01, 0.22)	0.23(0.10, 0.37)	−0.03(−0.25, 0.26)	0.19(0.13, 0.26)
	PM_2.5_	0.21(0.11, 0.31)	0.10(−0.02, 0.23)	0.31(0.14, 0.50)	0.05(−0.23, 0.45)	0.22(0.15, 0.30)
	SO_2_	0.16(0.04, 0.30)	0.13(−0.03, 0.32)	0.15(−0.03, 0.36)	−0.01(−0.36, 0.56)	0.15(0.06, 0.26)
	NO_2_	0.43(0.07, 0.90)	0.06(−0.29, 0.60)	0.75(0.10, 1.77)	−0.54(−0.11, 0.77)	0.45(0.16, 0.82)
	O_3_	0.10(−0.08, 0.31)	0.03(−0.21, 0.33)	0.21(−0.06, 0.56)	0.31(−0.22, 0.54)	0.14(0.01, 0.29)
Cold season	PM_10_	0.21(0.12, 0.31)	0.18(0.06, 0.32)	0.28(0.11, 0.46)	--	0.24(0.16, 0.32)
	PM_2.5_	0.24(0.13, 0.36)	0.20(0.06, 0.35)	0.33(0.13, 0.55)	--	0.26(0.17, 0.36)
	SO_2_	0.24(0.09, 0.41)	0.22(0.03, 0.44)	0.31(0.05, −0.62)	--	0.21(0.09, 0.34)
	NO_2_	1.09(0.45, 2.01)	0.73(0.08, 1.78)	1.97(0.63, 4.42)	--	1.21(0.65, 1.95)
	O_3_	−0.09(−0.38, 0.33)	−0.16(−0.47, 0.35)	0.02(−0.50, 1.10)	--	−0.01(−0.22, 0.27)
Warm season	PM_10_	0.06(−0.14, 0.30)	0.01(−0.26, 0.37)	0.15(−0.14, 0.56)	--	0.11(−0.04, 0.29)
	PM_2.5_	0.10(−0.14, 0.43)	−0.01(−0.32, 0.45)	0.25(−0.14, 0.82)	--	0.15(−0.06, 0.40)
	SO_2_	0.15(−0.28, 082)	0.92(−0.05, 2.89)	−0.33(−0.71, 0.57)	--	0.28(−0.10, 0.80)
	NO_2_	−0.39(−0.66, 0.10)	−0.28(−0.71, 0.83)	−0.37(−0.73, 0.45)	--	−0.30(−0.55, −0.08)
	O_3_	0.07(−0.15, 0.34)	−0.10(−0.38, 0.32)	0.19(−0.12, 0.61)	--	0.11(−0.05, 0.31)

The analyses were adjusted for seasonality, day of the week, temperature, relative humidity, air pressure, and wind speed

## Discussion

In this study, increased concentrations of outdoor air pollutants were associated with the increased mortality of acute lower respiratory tract infections among the general population of Shenyang, China. The effects of air pollution on the mortality of the acute lower respiratory tract infections were more significant in the cooler period. To our knowledge, this study was the first epidemiological analysis on the potentially harmful effects of air pollution on acute lower respiratory tract infection in northern China.

The 133 cases of non-accidental deaths in the study period revealed that PM_10_, PM_2.5_, SO_2_ and NO_2_ were related to the mortality of acute lower respiratory tract infections. In general, the associations between air pollution and mortality were more apparent in the elderly. This finding disagreed with the results of Gouveia et al. (18), where the relationship between air pollution and the number of deaths from daily acute lower respiratory tract infection was stronger in children (*<*5 years of age) than in those older than five years old. We observed that PM_10_, PM_2.5_, SO_2_, NO_2_ and O_3_ were more strongly associated with women than with men. This result contradicted that of Bates et al. (19). Nonetheless, previous studies have produced inconsistent results for air pollution and gender effects. For example, several studies have shown that PM_10_ is more closely linked to the male mortality rate of 20 males, whereas other studies have posited that women with airway reactivity more likely suffer from air pollution than men do (20, 21). However, the reasons for these discrepancies have yet to be clarified. Non-biological factors, such as poor socioeconomic status, poor education, and poor working conditions, may also contribute to the increased vulnerability of women.

Our results are mostly consistent with those of previous studies, which reported the considerably detrimental effects of particulate air pollution (22, 23). The estimated associations with PM (PM_10_ and PM_2.5_) markedly increased when gaseous pollutants (SO_2_ and NO_2_), particularly NO_2_, were added to the model. The estimates for the associations with NO_2_ were increased and PM_10_ and PM_2.5_ decrease when O_3_ were added. These results suggested that the relative effects of PM on gaseous pollutants and the relative effects of SO_2_ and NO_2_ differed from obtained by previous studies (24–26). Such differences may be due to the differences in other pollutant (e.g O_3_) concentrations, local meteorological conditions, and demographic characteristics of different populations (27–28).

Season was a factor that modified the associations between air pollution and health outcomes. We found significant seasonal variations in the effects of air pollution on the mortality of the acute lower respiratory tract infections. The estimated average in the cool period was significantly higher than that in the warm period, and the mean effect was limited during a season. The reasons for such seasonal differences are unclear but may be associated with the higher levels of contamination and the cool phase of the mortality of the acute lower respiratory tract infections.

Understanding the effects of exposure is critical to the development of public health policies, such as the winter heating policy, because of the serious air pollution in Shenyang, China. This study showed that high risk factors for acute exposure to outdoor air pollution may increase linearly without significant thresholds. Therefore, China’s air pollution-related burden may become its main public health problem. A larger population corresponds to a higher level of air pollution, implying that the impact may be greater than that in than high-income countries. Public health interventions may also be learned from our results. For example, during a cool season when the heating period or empty pollution is serious, patients with acute lower respiratory tract infection may opt to stay at home.

This study offers several advantages. First, this study utilized the scarcely used data in China’s northeastern region to assess air pollution and respiratory disease mortality, thus providing insight into the air pollution burden on the health of the population in the northeastern region. Second, this study well understood the adverse health effects of air pollution during the relevant heating period because the level of air pollution during the winter heating period in Shenyang exceeded the World Health Organization standard or the Chinese secondary standard (4, 29). Therefore, this study differs from previous studies (30) and can serve as a unique basis for China’s decision-making on air pollution control.

However, a possible limitation of this study is the actual level of population exposure to air pollution. First, we used modeling data rather than actual observations, and exposure measurement errors are unavoidable and have been shown to bias the estimates down (31–32). Thus, death may not be directly attributable to exposure to air pollutants (33–34). Second, as with most air pollution studies, exposure to air pollution is measured at the population rather than the personal level, and individual risk factors, such as smoking, alcohol, and underlying diseases, are unknown and uncontrollable in the analysis. Therefore, we cannot rule out the possibility of ecological bias and mixed effects of other risk factors of death. Thirdly, this study is based only on the Shenyang data, and air pollution associations may vary in different cities, suggesting that the results of this study should be considered in the local context.

## Conclusion

Time series analysis revealed that PM_10_, PM_2.5_, SO_2_ and NO_2_ were significantly high in women and the elderly in Shenyang, China. Our findings may help local decision makers to develop relevant air pollution control measures. Further large-scale studies are needed to confirm our results.

## Ethical considerations

Ethical issues (Including plagiarism, informed consent, misconduct, data fabrication and/or falsification, double publication and/or submission, redundancy, etc.) have been completely observed by the authors.
